# Arsenic and Diabetes: Navas-Acien et al. Respond

**DOI:** 10.1289/ehp.1206100R

**Published:** 2013-03-01

**Authors:** Ana Navas-Acien, Elizabeth A. Maull, Kristina A. Thayer

**Affiliations:** 1 Department of Environmental Health Sciences, Johns Hopkins Bloomberg School of Public Health, Baltimore, Maryland; 2 Division of the National Toxicology Program, National Institute of Environmental Health Sciences, National Institutes of Health; 3 Department of Health and Human Services, Research Triangle Park, North Carolina, E-mail: maull@niehs.nih.gov

The goal of the National Toxicology Program (NTP) workshop review was to comprehensively evaluate the association between arsenic and diabetes, including epidemiologic and experimental evidence (Maull et al. 2012). Members of the arsenic breakout group carefully evaluated differences in methodologic approaches used to analyze general population studies, including NHANES (National Health Nutrition and Examination Survey) studies, trying to understand the biology and technical limitations of biomarkers of inorganic arsenic exposure measured in urine, as well as their implications for study findings.

In his letter, Smith presents his arguments in a selective manner, overlooking important evidence and facts. First, multiple studies included in the NTP workshop review [see our Table 2 (Maull et al. 2012)] support the relationship of low-to-moderate arsenic exposure levels (< 150 µg/L in drinking water) with diabetes and diabetes-related end points. Second, when indicating that subtracting arsenobetaine from total arsenic is the recommended method to evaluate inorganic arsenic exposure, Smith ignored research conducted in the last decade showing that other seafood arsenicals (arsenosugars, arsenolipids) also contribute to total urinary arsenic (European Food Safety Authority 2009; Francesconi et al. 2002; Maull et al. 2012). Subtracting arsenobetaine from total arsenic is insufficient to eliminate the contribution of seafood arsenicals in populations where seafood is common (see Figure 1 of Maull et al. 2012). Third, Smith criticized the adjustment of the association between total urinary arsenic and diabetes for arsenobetaine without mentioning that total urinary arsenic was associated with diabetes without adjusting for arsenobetaine in NHANES participants with very low or undetectable arsenobetaine (Navas-Acien et al. 2008, 2009), populations where total urinary arsenic likely reflects inorganic arsenic exposure. These results at low arsenobetaine concentrations exclude collinearity as an explanation for the findings. The consistency between analyses that are restricted to very low arsenobetaine concentrations and analyses that statistically adjust for arsenobetaine is not a surprise because both epidemiologic strategies are able to minimize the contribution of other seafood arsenicals to total urine arsenic concentrations. In a transparent manner, the NTP workshop review acknowledged the differing interpretations of the NHANES studies, concluding that the

lack of consistency… warrants caution in interpreting results and highlights the importance of having good analytical methods to distinguish inorganic arsenic.

As summarized in our NTP workshop review (Maull et al. 2012), the evidence is currently insufficient to conclude that arsenic is associated with diabetes at low-to-moderate exposure levels. Limitations of many of the available studies included the lack of prospective evidence, limitations in exposure and outcome assessment, and lack of adjustment for appropriate confounders. Since the publication of the NTP workshop review, additional cross-sectional (Gribble et al. 2012) and prospective (James et al. 2012; Kim et al., in press) studies conducted in the United States and supporting the association between arsenic and diabetes have been published.

Millions of Americans are exposed to arsenic through drinking water and food. Smith recommended that arsenic research focus on levels in drinking water that are 15 times higher than the current safety standards of the World Health Organization, U.S. Environmental Protection Agency, and European Union. In our opinion, research and public health efforts should focus on preventing arsenic exposure. At low-to-moderate levels, state-of-the-art epidemiologic tools—including cost-effective designs, high quality exposure and outcome assessment, careful evaluation of dose–response relationships, and integrated methods to evaluate gene–environment interactions and mechanistic pathways—can provide insight into the health effects of arsenic exposure through drinking water and food.
